# Diverse Taxonomies for Diverse Chemistries: Enhanced Representation of Natural Product Metabolism in UniProtKB

**DOI:** 10.3390/metabo11010048

**Published:** 2021-01-12

**Authors:** Marc Feuermann, Emmanuel Boutet, Anne Morgat, Kristian B. Axelsen, Parit Bansal, Jerven Bolleman, Edouard de Castro, Elisabeth Coudert, Elisabeth Gasteiger, Sébastien Géhant, Damien Lieberherr, Thierry Lombardot, Teresa B. Neto, Ivo Pedruzzi, Sylvain Poux, Monica Pozzato, Nicole Redaschi, Alan Bridge, on behalf of the UniProt Consortium

**Affiliations:** 1 Swiss-Prot Group, SIB Swiss Institute of Bioinformatics, CMU, 1 Michel-Servet, CH-1211 Geneva 4, Switzerland; Anne.Morgat@sib.swiss (A.M.); Kristian.Axelsen@sib.swiss (K.B.A.); Parit.Bansal@sib.swiss (P.B.); Jerven.Bolleman@sib.swiss (J.B.); Edouard.deCastro@sib.swiss (E.d.C.); Elisabeth.Coudert@sib.swiss (E.C.); Elisabeth.Gasteiger@sib.swiss (E.G.); Sebastien.Gehant@sib.swiss (S.G.); Damien.Lieberherr@sib.swiss (D.L.); Thierry.Lombardot@unil.ch (T.L.); Teresa.Neto@sib.swiss (T.B.N.); Ivo.Pedruzzi@sib.swiss (I.P.); Sylvain.Poux@sib.swiss (S.P.); Monica.Pozzato@sib.swiss (M.P.); Nicole.Redaschi@sib.swiss (N.R.); Alan.Bridge@sib.swiss (A.B.);; 2 European Molecular Biology Laboratory, European Bioinformatics Institute (EMBL-EBI), Wellcome Trust Genome Campus, Hinxton, Cambridge CB10 1SD, UK; 3 Protein Information Resource, University of Delaware, 15 Innovation Way, Suite 205, Newark, DE 19711, USA; 4 Protein Information Resource, Georgetown University Medical Center, 3300 Whitehaven Street NorthWest, Suite 1200, Washington, DC 20007, USA

**Keywords:** natural product, enzyme, biochemical reaction, biocuration, ontology, knowledge base, RDF, SPARQL, semantic web, cheminformatics

## Abstract

The UniProt Knowledgebase UniProtKB is a comprehensive, high-quality, and freely accessible resource of protein sequences and functional annotation that covers genomes and proteomes from tens of thousands of taxa, including a broad range of plants and microorganisms producing natural products of medical, nutritional, and agronomical interest. Here we describe work that enhances the utility of UniProtKB as a support for both the study of natural products and for their discovery. The foundation of this work is an improved representation of natural product metabolism in UniProtKB using Rhea, an expert-curated knowledgebase of biochemical reactions, that is built on the ChEBI (Chemical Entities of Biological Interest) ontology of small molecules. Knowledge of natural products and precursors is captured in ChEBI, enzyme-catalyzed reactions in Rhea, and enzymes in UniProtKB/Swiss-Prot, thereby linking chemical structure data directly to protein knowledge. We provide a practical demonstration of how users can search UniProtKB for protein knowledge relevant to natural products through interactive or programmatic queries using metabolite names and synonyms, chemical identifiers, chemical classes, and chemical structures and show how to federate UniProtKB with other data and knowledge resources and tools using semantic web technologies such as RDF and SPARQL. All UniProtKB data are freely available for download in a broad range of formats for users to further mine or exploit as an annotation source, to enrich other natural product datasets and databases.

## 1. Introduction

Bacteria, fungi, and plants produce an enormous variety of natural products (NPs), secondary metabolites with a fantastic array of molecular structures and biological and pharmaceutical properties. Natural products are a rich source for drugs—including anticancer, antibiotic, and antifungal therapies—as well as biofuels, cosmetics, perfumes, and flavors, to cite but a few applications [1,2,3].

**Figure 1:**
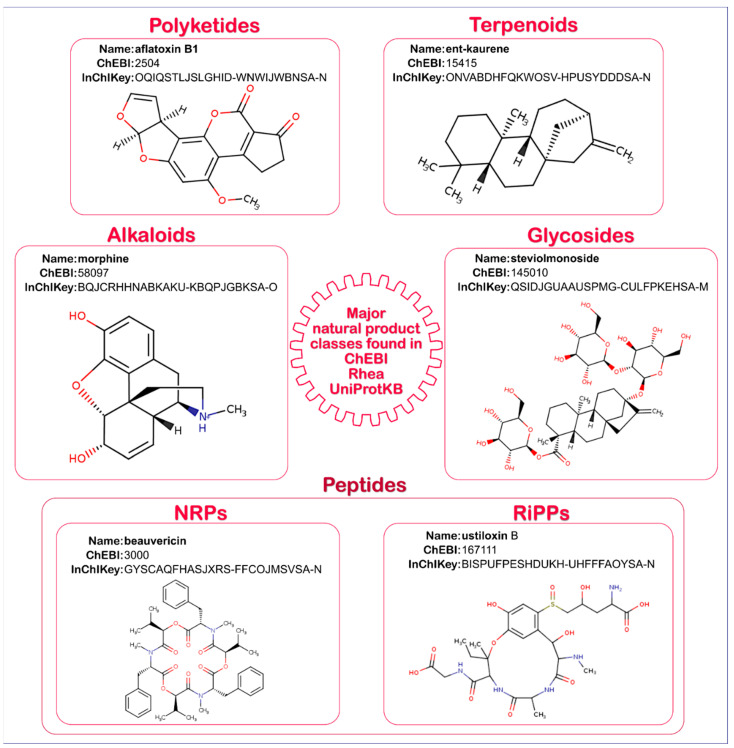
The major classes of natural products and representatives found in Chemical Entities of Biological Interest (ChEBI), Rhea, and UniProtKB. Examples used are ent-kaurene for terpenoids, morphine for alkaloids, steviolmonoside for glycosides, aflatoxin B1 for polyketides, beauvericin for non-ribosomal peptides (NRPs), and ustoloxin B for ribosomally synthesized and post-translationally modified peptides (RiPPs) starting with the precursor ribosomally synthesized cyclic peptide ustiloxin B precursor ustA (UniProtKB: B8NM66).

Most classes of natural products (Figure 1), including terpenoids [1], alkaloids [4], glycosides [5,6], and polyketides [7], as well as non-ribosomal peptides (NRPs) [8], are synthesized by complex enzymatic pathways. The exceptions to this rule are the ribosomally synthesized and post-translationally modified peptides (RiPPs), which are encoded directly by the genome and synthesized via the classical transcription/translation machinery [9]. The genes coding for these pathways are mainly organized in co-regulated (and generally transcriptionally repressed) biosynthetic gene clusters (BGCs) [10,11], which also encode the necessary transcription factors, transporters [12,13], and proteins that protect against the toxic effects of natural products in their “hosts” [14].

Our knowledge of the genomic organization, biology, and chemistry of natural product biosynthetic pathways is rapidly increasing, thanks to advances in experimental and computational approaches and to the development of open-access databases and tools. These include reference resources of natural product structures such as The Natural Products Atlas [15] and the COlleCtion of Open NatUral producTs (COCONUT) [16,17], as well as tools to catalog, identify, and annotate BGCs in microbial and plant genomes such as the Integrated Microbial Genomes Atlas of Biosynthetic gene Clusters (IMG-ABC) [18], ClusterMine360, a database of microbial polyketide and non-ribosomal peptide gene clusters [19], MIBiG [13], which provides a minimum information specification for BGCs, and antiSMASH (antibiotics and Secondary Metabolite Analysis Shell) [20] and plantiSMASH [21], which exploit knowledge of the domain composition of key enzymes in BGCs to predict the natural products associated with each BGC.

All genome data mining approaches to natural product discovery ultimately depend on the correct annotation of experimentally characterized enzymes, which provides the crucial link from genome sequence to chemical structure. The UniProt Knowledgebase (UniProtKB, www.uniprot.org) is one source of such knowledge—a reference resource of protein sequences and functional annotation that is widely used for functional analyses of genomic, transcriptomic, and proteomic data [22]. We have recently extended the scope of UniProtKB to cover small molecule metabolite and metabolomic data, including but not limited to natural products. We have performed a complete reannotation of all enzyme data in UniProtKB [23] using Rhea (www.rhea-db.org) [24], a freely available resource of expert-curated biochemical and transport reactions described using the Chemical Entities of Biological Interest (ChEBI) ontology of small molecules (www.ebi.ac.uk/chebi) [25], and have begun a new program of annotation of natural products that focuses on enzymes in well-characterized pathways and the reactions they catalyze. We have also developed a range of tools and services that allow users of UniProtKB to map small molecule metabolites to proteins, facilitating the integration of metabolomics and proteomics. In the following section, we describe the annotation of enzymes and natural products in UniProtKB and provide examples of how to query UniProtKB for this knowledge using interactive and programmatic means.

## 2. Results

### 2.1. Natural Product Annotation in UniProtKB

**Figure 2:**
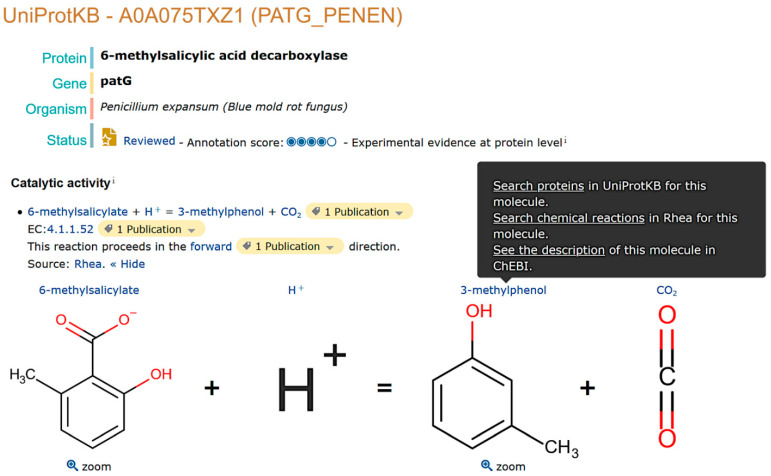
Enzyme annotation in UniProtKB/Swiss-Prot. The figure highlights the reaction catalyzed by the 6-methylsalicylic acid decarboxylase (patG) of *Penicillium expansum* (UniProtKB: A0A075TXZ1). The reaction is evidenced by a publication and EC 4.1.1.52 (enzyme class). Hover over the name of a reaction participant to display a tooltip allowing navigation between Rhea, ChEBI, and UniProt resources.

UniProtKB now uses the Rhea knowledgebase of biochemical transformations and transport reactions, itself built on the chemical ontology ChEBI, as the standard for the annotation of enzymes and transporters (Figure 2) [23]. Rhea and ChEBI provide computationally tractable knowledge of small molecule metabolites and their transformations and movements in UniProtKB-facilitating metabolomic data integration with that from proteomics, transcriptomics, and genomics, as well as a broad range of data mining operations, some of which we will see later. At the time of writing (UniProtKB release 2020_06 of December 2020), UniProtKB/Swiss-Prot, the curated section of UniProtKB (see Section 4.2 and Section 4.3), contains 220,656 enzyme sequences annotated with Rhea reactions, while UniProtKB/TrEMBL, the unreviewed section of UniProtKB annotated using computational means, contains more than 22 million enzyme sequences linked to Rhea reactions (as described in Section 4.4). Enzyme annotations in UniProtKB as a whole cover 8708 unique Rhea reactions involving 8022 unique ChEBI compounds. Enzyme annotation in UniProtKB, like all curation, is an ongoing process with new data released every two months. Because of the biological and pharmaceutical potential of natural products as well as the interest expressed by the NP community, we are working to improve the coverage of natural product metabolism in UniProtKB using Rhea and recently launched a dedicated annotation program that focuses specifically on secondary metabolism in fungi and plants.

ChEBI is a chemical ontology of a broad scope that includes both primary metabolites and secondary metabolites or natural products. To assess the coverage of natural products in ChEBI, Rhea, and UniProtKB, we compared all chemical structures in ChEBI to those in two reference resources of natural products, The Natural Product Atlas (www.npatlas.org) [15] and COCONUT (coconut.naturalproducts.net) [16,17]. We mapped chemical structures from ChEBI to The Natural Product Atlas and COCONUT using their InChIKeys—a hash representation of the chemical structure that is particularly suited for chemical structure searching [26] (see Section 2.2.1 for more on InChIKeys, which can also be used to search UniProtKB). Of the 8022 unique ChEBI compounds used in UniProtKB-Rhea annotations, 1052 ChEBI entries (13% of the total) are found in either The Natural Product Atlas or COCONUT or both. In other words, we have linked 1052 presumed natural products from ChEBI to their cognate enzymes in UniProtKB to date. The Natural Products Atlas clusters compounds that share very high structural similarity, providing a way to quickly find other compounds that are closely related to a given compound of interest. Of the 1052 natural products from ChEBI that are curated in UniProtKB/Swiss-Prot, 261 are mapped to 171 clusters of natural product structures in The Natural Products Atlas: these clusters include a total of 4542 natural product structures. This defines a possible upper bound on the number of relevant enzyme-natural product structure links that can be derived using UniProtKB/Swiss-Prot and The Natural Products Atlas at the current time. ChEBI release 191 of September 2020 includes a total of 105,802 ChEBI entries with fully defined structures, of which 11,422 ChEBI entries (10.7% of the total) are found in either The Natural Product Atlas or COCONUT or both. UniProtKB/Swiss-Prot annotations currently cover around 10% of these, thus there is clearly great scope to expand the coverage of natural product structures in ChEBI, Rhea, and UniProtKB; some strategies for doing so are discussed in Section 3, “Conclusions and Perspectives”.

### 2.2. Data Access

In this section, we look at ways to access protein knowledge relating to natural products either interactively, using the UniProt website, or programmatically, using the UniProt REST API or SPARQL endpoint.

#### 2.2.1. The UniProt Website

**Figure 3:**
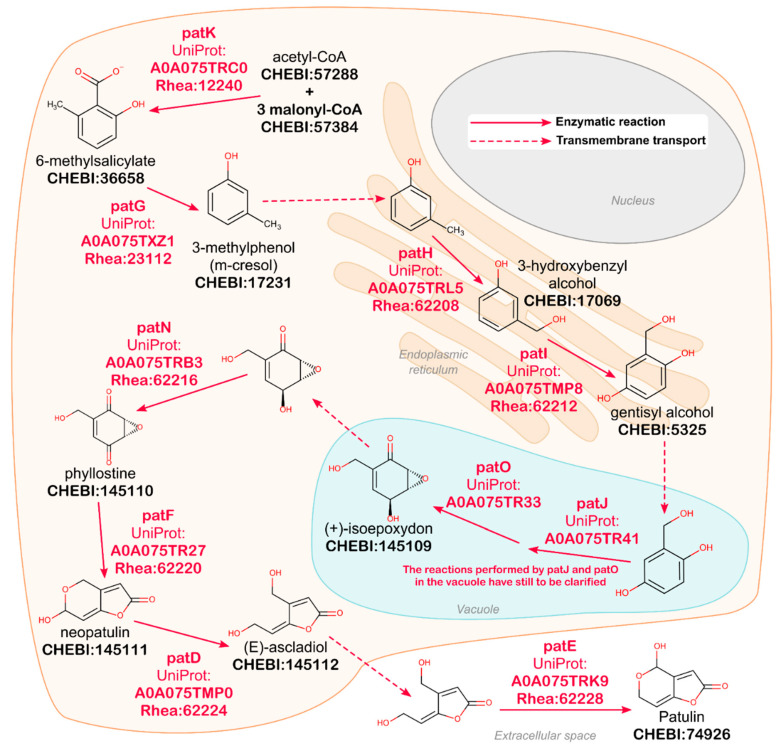
Curation of the patulin biosynthetic pathway in *Penicillium expansum*. The figure shows a schematic representation of the patulin biosynthesis pathway [28], which was fully curated in UniProtKB/Swiss-Prot. This pathway map was reconstructed using the 2D structures from ChEBI, the reactions provided by Rhea and their corresponding enzymes as annotated in UniProtKB (identifiers for each are indicated). UniProtKB/Swiss-Prot also provides additional data such as the subcellar location of each protein when known. The solid arrows indicate enzymatic reactions; the dashed arrows indicate transport reactions. The subcellular location of the enzymes illustrates the importance of compartmentalization for natural product biosynthesis [28]. All UniProtKB entry proteins involved in the patulin biosynthesis pathway can be retrieved using the following URL: www.uniprot.org/uniprot/?query=patulin&fil=organism%3A%22Penicillium+expansum+%28Blue+mold+rot+fungus%29+%5B27334%5D%22+AND+reviewed%3Ayes.

The UniProt website (www.uniprot.org) constitutes the main point of entry for most UniProt users and provides a range of simple and advanced search options [23,27]. We demonstrate some of these options using examples from the patulin biosynthetic pathway (Figure 3). Patulin is an acetate-derived tetraketide mycotoxin produced by *Penicillium expansum* and several related fungal species. It is the most common mycotoxin found in apples and apple-derived products and shows antimicrobial properties against several bacteria. The patulin BGC from *Penicillium expansum* is composed of 15 genes encoding 11 enzymes, three transporters, and one BGC-specific transcription factor [28]. Its biosynthesis is quite well studied, and all relevant information has been captured in UniProtKB, Rhea, and ChEBI through expert curation.

The easiest way to search UniProt is to use the simple search tool, which accepts text (including natural language or identifiers) and boolean operators. A query with a single term—patulin—returns a list of all UniProtKB protein sequence records containing this word in any annotation field or topic(s) (Figure S1). One way to limit the search to proteins that actually interact with or metabolize patulin itself is to search using the chemical structure of patulin encoded as an InChIKey, a hash representation of a chemical structure that is composed of three blocks [26]. These can be understood using the InChIKey for patulin, which is ZRWPUFFVAOMMNM-UHFFFAOYSA-N; the first block of 14 characters (ZRWPUFFVAOMMNM) encodes information on connectivity, the second block of 10 characters (UHFFFAOYSA) encodes information on stereochemistry, and the third block of one character (N) encodes information on charge (N for neutral, M for −1, O for +1, and so on). A more complete description of InChIKey is available at www.inchi-trust.org. Chemical structure searches in UniProtKB can be performed with full or partial InChIKey—either the first block only (ZRWPUFFVAOMMNM for patulin) or both the first and second blocks (ZRWPUFFVAOMMNM-UHFFFAOYSA). These options allow users to deal with ambiguity surrounding stereochemistry and charge states, respectively.

A second way to limit the search to proteins that actually interact with or metabolize patulin itself is to search using the chemical identifier for patulin from ChEBI (which is CHEBI:74926). Chemical identifier searches leverage the relationships encoded in the ChEBI ontology, allowing users to expand their search to include all members of defined chemical classes, such as the gamma lactones (CHEBI:37581), of which patulin is one member. Note that ChEBI assigns a distinct identifier to each charge state of a given structure; UniProt deals with this by mapping each ChEBI identifier searched to that of the major microspecies at pH 7.3, which is the form used in Rhea and UniProtKB annotations. This mapping is performed during the search, using a mapping file provided by Rhea (at www.rhea-db.org/help/download). Further information about chemical data search in UniProtKB can be found in our online documentation at www.uniprot.org/help/chemical_data_search.

**Figure 4:**
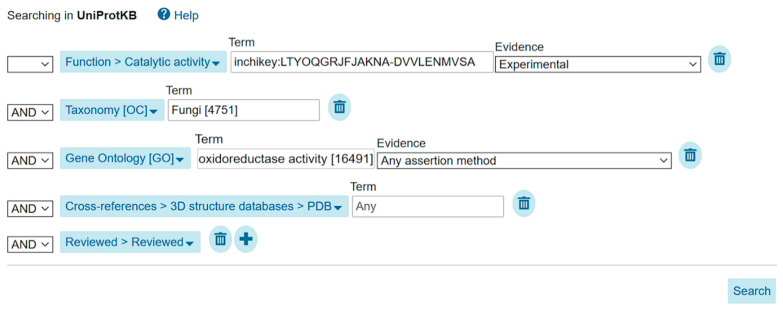
Sample query using the UniProt website advanced search tool to retrieve, in UniProtKB/Swiss-Prot, fungal oxidoreductases that metabolize malonyl-CoA, with published 3D structure(s). The query retrieves expert-curated (Field: Reviewed > Reviewed) oxidoreductases (Field: Gene Ontology [GO], Term: “oxidoreductase activity [16491]”) of fungal origin (Field: Taxonomy, Term: “Fungi [4751]”) metabolizing (Field: Function > Catalytic activity, Term: “inchikey: LTYOQGRJFJAKNA-DVVLENMVSA”) and for which protein 3D structure data are available (Field: Cross-references > 3D structure databases > PDB).

Users can refine searches by applying suggested filters (Figures S2 and S3), personalize the content displayed in columns (Figures S4 and S5), and download all or selected matched protein entries in a variety of formats (Figure S6) including XML, RDF/XML or text (for UniProt entries), FASTA (for UniProt sequences), tab-delimited or excel table (for personalized result lists), and GFF (for sequence features). Another way to perform more fine-grained searches is by using our advanced query builder (accessed from the front page), which allows users to define search fields for terms, to link search term/field combinations using boolean operators, and to specify the level of evidence required for a match—such as requiring experimental evidence for some annotation. Figure 4 shows the advanced search tool on www.uniprot.org being used to build a query for fungal oxidoreductases that are proven to metabolize malonyl-CoA, a precursor of many polyketide natural products including patulin, and for which protein structure data are available. We search for malonyl-CoA using the first two layers of the InChIKey (LTYOQGRJFJAKNA-DVVLENMVSA, thereby disregarding charge), specify the fungal taxon using the appropriate identifier from the NCBI taxonomy (TaxID:4751), oxidoreductase function using the Gene Ontology (GO:0016491), and the required cross-reference to the PDB (without additional metadata).

#### 2.2.2. Programmatic Access to UniProt-REST API

The UniProt website has RESTful URLs that can be bookmarked, linked, and used in programs for all entries, queries, and tools available through the website (see details at www.uniprot.org/help/programmatic_access). Table 1 provides the URLs for each of the queries described in the preceding section.

#### 2.2.3. Programmatic Access to UniProt-SPARQL

All UniProt data are available in RDF, a core semantic web technology for the World Wide Web Consortium that is well suited to applications in distributed environments (see www.w3.org/RDF/ for more details). The UniProt SPARQL endpoint (sparql.uniprot.org/sparql) allows users to perform complex queries on UniProt RDF data and to combine UniProt RDF data in real time with RDF data from other resources providing SPARQL endpoints, through so-called “federated queries”. Resources that provide SPARQL endpoints that may be of particular interest in natural product research and that are highly complementary to UniProt, include Rhea [23,24], the Integrated Database of Small Molecules (IDSM) [29], which supports chemical similarity and chemical substructure searches over ChEBI and other chemical structure databases, the OMA [30] and OrthoDB [31] resources of orthologous groups, and the MetaNetX resource of genome-scale metabolic models [32]. A tutorial for querying these resources with SPARQL is available at edu.sib.swiss/course/view.php?id=440.

**Figure 5:**
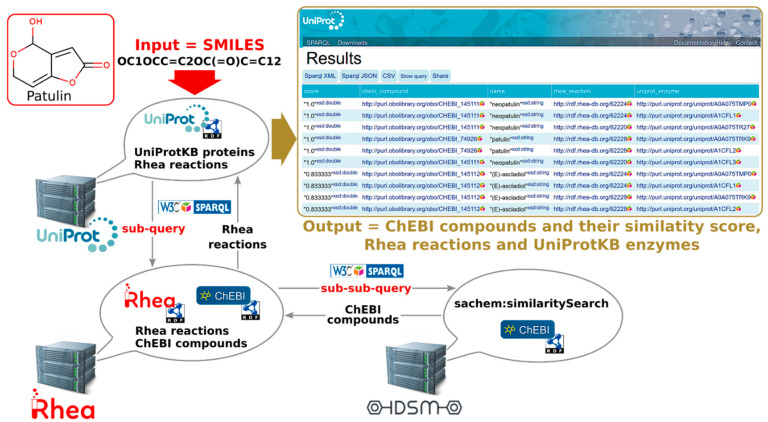
Graphical representation of the sample federated SPARQL query displayed in Figure S7 and its results. The query is performed at the UniProt SPARQL endpoint, which first “calls” the Rhea SPARQL endpoint, which itself “calls” the IDSM SPARQL endpoint. The actual compound similarity search (sachem:similaritySearch) is performed by the IDSM endpoint, which returns ChEBI compounds identical or similar to patulin to the Rhea endpoint. The Rhea endpoint then assembles a list of matching reactions and passes this list back to the UniProt endpoint, which finally maps the reactions to all possible enzymes and creates the desired result set of cognate chemicals, reactions, and enzymes. The results are available at tinyurl.com/sparql-uniprot.

We illustrate some of the advantages of SPARQL using a sample federated query that uses the UniProt, Rhea, and IDSM SPARQL endpoints to perform chemical similarity searches, extending the simple InChIKey-based structure searches that are possible using the UniProt website and REST API (Figure 5 and Figure S7). Note that chemical similarity searching is not supported by the UniProt website or REST API-IDSM and Rhea “lend” this functionality on the fly to the UniProt SPARQL endpoint, through query federation, allowing an end-user of the system to act as if they are using a single integrated database. The query shown in Figure S7 will retrieve all enzymes annotated in UniProtKB/Swiss-Prot that metabolize compounds similar to patulin—but not necessarily identical to patulin. The query uses the SMILES representation (Simplified Molecular-Input Line-Entry System) (opensmiles.org) of patulin, as required for structure searches by IDSM, and uses the sachem:similaritySearch procedure call pattern developed by the IDSM team [33] with a similarity score threshold of 0.8 (the similarity score is based on Jaccard similarity of Morgan-style connectivity fingerprints). The query is designed with two nested services (“calls”) as illustrated in Figure 5. It is run from the UniProt SPARQL endpoint, which first “calls” the Rhea SPARQL endpoint, which itself “calls” the IDSM SPARQL endpoint. The results of this query are available at tinyurl.com/sparql-uniprot and include enzymes that metabolize neopatulin (CHEBI:145111), such as patD and patF, and (*E*)-ascladiol (CHEBI:145112), such as patD and patE. To see SPARQL in action simply copy-paste the query shown in Figure S7 into sparql.uniprot.org/sparql and run it.

## 3. Discussion

UniProt is engaged in a continuous effort to improve the integration, uniformization, sharing, and representation of protein knowledge. UniProtKB now uses the Rhea knowledgebase of biochemical transformations and transport reactions, based on the chemical ontology ChEBI, to link enzymes and transporters to explicit representations of the chemical structures of their substrates and products. Through Rhea and ChEBI, UniProtKB provides a platform to integrate knowledge of small molecule metabolites, including but not limited to natural products, with knowledge of protein sequences and their functions in a broad range of species. Expert-curated knowledge of protein function in UniProtKB/Swiss-Prot is complemented by additional knowledge and links from a wide network of collaborating resources covering many aspects of protein biology, including InterPro (protein domains and families) [34], PDBe (protein structures) [35], Reactome (pathways) [36], the IMEx databases of molecular interactions [37], the Gene Ontology Consortium (additional GO annotations) [38,39], PubMed literature (pubmed.ncbi.nlm.nih.gov), and many more. Researchers in the field of natural products can leverage these additional annotations, links, and integrated datasets to study a broad array of aspects of protein biology. The Kyoto Encyclopedia of Genes and Genomes database (KEGG) provides a collection of manually drawn pathway maps including some secondary metabolism pathways (www.kegg.jp/kegg/pathway.html) [40,41,42]. Our work does not directly supply such maps but provides all the information to users to reconstruct similar pathway diagrams (see Figure 3 as an example) by using the 2D structures from ChEBI, the reactions provided by Rhea, and their corresponding enzymes as annotated in UniProtKB. 

The curation of natural product enzymes at UniProt is a new activity, begun after the introduction of Rhea as the standard for enzyme annotation in UniProtKB in late 2018 [23]. The focus of this annotation program—capturing the links between enzymes and natural products throughout pathways—complements the efforts of specialist resources such as The Natural Product Atlas [15] and COCONUT [16,17], which provide comprehensive libraries of all known natural products and of resources and tools that aim to provide comprehensive annotation of biosynthetic gene clusters (BGCs) and their ultimate products—such as antiSMASH [20], ClusterMine360 [19], and BiGFAM [43], which provide comprehensive annotation of biosynthetic gene clusters (BGCs) and their ultimate products.

Current work at UniProt and Rhea focuses on improving the coverage and representation of natural products in ChEBI, reactions in Rhea, and enzymes in UniProtKB. UniProtKB currently provides enzyme annotations for over 1000 distinct natural products from ChEBI (see Section 2.1). While this is only a small fraction of the vast space of natural product structures that exist in specialist resources such as COCONUT and the Natural Product Atlas, knowledge of enzymatic pathways is not available for many if not most of the natural products found in these resources. Our own initial survey of literature cited in The Natural Products Atlas suggests that only around 10% of the literature cited deals with enzymes, while the majority describes the purification and characterization of the natural products themselves; we plan to further screen this subset of relevant literature in the Natural Product Atlas for enzyme annotations in UniProtKB. Another focus of our curation efforts is to improve the coverage of biosynthetic pathways in the Biological Process branch of the Gene Ontology. These biosynthetic pathway definitions provide a natural bridge between individual enzymes and biosynthetic gene clusters; the individual steps of pathways, captured in Rhea reactions, are also being mapped to the GO Molecular Function branch (Harold Drabkin, Peter d’Eustachio, Chris Mungall, and Paul Thomas, unpublished work). We also plan to expand the scope of Rhea to capture transformations—pairs of compounds that are known to be linked in or by reactions, but for which precise mechanistic detail on how the reaction occurs is not yet available (see goldbook.iupac.org/terms/view/T06446). Capturing transformations will further enhance our coverage of natural product biosynthetic pathways and other types too, including pathways for the modification of environmental pollutants [44,45].

## 4. Materials and Methods

We begin with a brief overview of the contents of UniProtKB, including the sources of protein sequences, how sequences are organized and classified, and how they are enriched with functional annotation—either by expert curators or using computational approaches. We highlight some particular areas of interest for users studying natural products and the enzymes that produce them, before describing in more detail how knowledge of natural products and their enzymes is captured in UniProtKB.

### 4.1. Protein Sequences in UniProtKB

UniProtKB provides broad coverage of protein sequence space, incorporating protein sequence data translated from International Nucleotide Sequence Database Consortium INSDC (composed of EMBL, GenBank, and DDBJ) [46], from Ensembl [47] and Ensembl Genomes [48], and other resources such as the Protein Data Bank in Europe PDBe [35]. UniProt proteomes group the protein sequence records of a complete genome into a single set, which can be easily downloaded for further analysis (see www.uniprot.org/proteomes).

### 4.2. UniProtKB Sections

UniProtKB is composed of two sections: UniProtKB/Swiss-Prot, the reviewed section of UniProtKB, which contains protein sequence records enriched with human and machine-readable information extracted from the literature by expert curators as well as curator-evaluated computational analysis, and UniProtKB/TrEMBL, the unreviewed section of UniProtKB, which contains protein sequence records annotated by automated systems. At the time of writing (UniProt release 2020_06 of December 2020), UniProtKB/Swiss-Prot contains 563,972 protein sequence records from 13,984 taxa, enriched with information from over 240,000 publications, and UniProtKB/TrEMBL 209,157,139 protein sequence records from 1,233,899 taxa (www.uniprot.org/statistics).

### 4.3. Expert Curation in UniProtKB/Swiss-Prot

Expert curation in UniProtKB/Swiss-Prot captures many aspects of protein knowledge in forms that both humans and machines can understand and reason over-using human-readable text, controlled vocabularies, and ontologies. These include protein functions, subcellular locations, interactions, expression patterns, involvement in disease, and a broad range of sequence features, including active sites, ligand binding sites, post-translational modifications (PTMs), and experimentally induced mutations and naturally occurring variations with functional impact (Figure S8).

Enzyme and transporter function is of particular interest for studies of natural products and is described in UniProtKB using Rhea (www.rhea-db.org) [23,24], a comprehensive expert-curated knowledgebase of biochemical transformations and transport reactions described using the ChEBI (Chemical Entities of Biological Interest) ontology (www.ebi.ac.uk/chebi) of small molecules [24]. Rhea provides machine-readable descriptions of over 13,000 biochemical transformations and transport reactions sourced from over 14,000 literature citations. It covers reactions described by the Enzyme Commission of the IUBMB (EC numbers) and thousands of more additional reactions not covered by EC numbers. We provide some examples of how Rhea annotations can be leveraged to integrate the knowledge of small molecule metabolites and proteins in Section 2.2, “Data Access”. In addition to Rhea, UniProtKB also provides enzyme annotations using the enzyme classification of the IUBMB (EC numbers) and using other reference vocabularies and ontologies such as the Gene Ontology [38,39], which covers molecular functions, biological processes (pathways), and cellular components or subcellular locations. Compartmentalization of cellular biochemistry plays a critical role in natural product biosynthesis, although our knowledge for many enzymes and transporters is lacking [28,49] (Figure 3). Enzyme cofactors are also annotated and, like reaction participants in Rhea, mapped to the ChEBI ontology. Kinetic parameters (Km, Vmax) and information on the biophysical and chemical properties of enzymes such as optimal pH and temperature, redox potential, or maximal absorption of photoreactive proteins are also reported when available. Functional features such as active sites, substrate binding sites, ligand and cofactor binding sites, sites of experimental mutagenesis, and PTMs are curated from the literature and mined by careful analysis of 3D protein structures from PDBe. Like reaction participants and cofactors, modified residues resulting from PTMs (www.uniprot.org/help/mod_res) are also mapped to the ChEBI ontology (www.uniprot.org/docs/ptmlist), as these modified residues can be considered as groups derived from small molecules. Additionally, we are mapping all small molecule ligands mentioned in UniProtKB feature descriptions to ChEBI. The release of this dataset, planned for late 2021, will complete the mapping of all small molecule data in UniProtKB to ChEBI.

Finally, it is worth noting that all protein sequences in UniProtKB/Swiss-Prot are themselves checked and where necessary corrected. Curators check each sequence for errors such as frameshifts, as well as for erroneous predictions causing gene fusions, splits, and splicing errors, and flag all sequences that are corrected (complete documentation at www.uniprot.org/help/sequence_caution). Around 8% of all protein sequence records in UniProtKB/Swiss-Prot required some form of manual correction.

### 4.4. Automated Annotation in UniProtKB/TrEMBL

UniProtKB/TrEMBL, the unreviewed component of UniProtKB, is annotated by two rule-based annotation systems (see www.uniprot.org/help/automatic_annotation): UniRule, which consists of expert-curated rules (see www.uniprot.org/unirule/), and ARBA, which consists of automatically generated rules (see www.uniprot.org/help/arba) [50]. Both systems use experimentally characterized proteins in UniProtKB/Swiss-Prot as the template to create rules that specify relevant annotations and the conditions which must be satisfied for those annotations to apply—such as family membership and the presence of key functional residues such as active sites. Together UniRule and ARBA cover around 112 million protein sequences in UniProtKB/TrEMBL, about 50% of all sequences in this section. We also provide an encoding of a subset of UniRules as SPARQL queries (see Section 2.2.3), which allows the application of our rules for genome and proteome annotation using any off the shelf SPARQL query engine [51].

### 4.5. Evidence for and Provenance of Annotations in UniProtKB/Swiss-Prot and UniProtKB/TrEMBL

Evidence and provenance for expert-curated and automatically generated annotations in UniProtKB/Swiss-Prot and UniProtKB/TrEMBL are captured using “evidence tags” consisting of two parts: a mandatory evidence “type” drawn from the Evidence and Conclusion Ontology (ECO, www.evidenceontology.org/ [52]) and an optional “source”, such as a literature reference or a record from another database, such as PDBe (see www.uniprot.org/help/evidences).

### 4.6. Protein Sequence Classification in UniProtKB

Protein family membership and domain composition are useful predictors of enzyme specificity for chemical-class-defining enzymes such as polyketide synthases (PKSs) or non-ribosomal peptide synthetases (NRPSs) and are widely used in genome data mining approaches for the identification of BGCs. All sequences in UniProtKB/Swiss-Prot and UniProtKB/TrEMBL are annotated using InterProScan from InterPro [34], a powerful integrated database and diagnostic tool that uses predictive models of domains, families, and other features such as protein disorder from a broad range of providers (see www.ebi.ac.uk/interpro/about/consortium). UniProtKB curators also capture the knowledge of new protein families reported in the literature but not yet covered by InterPro contributors, such as thirteen new families linked to natural product biosynthesis (Table S1). Twelve of these families contain experimentally characterized enzymes while one, the ltmS family (named after the Ltms protein from the fungus *Epichloe festucae*), contains an as yet uncharacterized protein involved in lolitrem biosynthesis that is conserved in all indole-diterpene producers [53].

### 4.7. UniProtKB as A Hub

UniProtKB provides links to over 100 reference knowledgebases and data repositories, links that are exploited in our powerful identifier mapping and batch retrieval tool (www.uniprot.org/uploadlists). UniProtKB also integrates complementary data from many of these linked resources, including the aforementioned protein classifications from InterPro as well as others such as molecular interaction data from IMEx database [37], peptide data from ProteomeXchange database [54], and variation data from a range of clinical resources including ClinVar [55], COSMIC [56], 1000 Genomes [57], and dbSNP [58].

## Figures and Tables

**Table 1:** Examples of queries on www.uniprot.org and the corresponding URLs (that can be bookmarked for future reference).

Query on UniProtKB	URL
patulin, by name	uniprot.org/uniprot/?query=patulin
patulin, by structure (using the first two blocks of the InChIKey)	uniprot.org/uniprot/?query=inchikey:ZRWPUFFVAOMMNM-UHFFFAOYSA
patulin, by chemical identifier (CHEBI:74926)	uniprot.org/uniprot/?query=%09CHEBI%3A74926
all members of the class gamma lactone, by chemical identifier (CHEBI:37581)	uniprot.org/uniprot/?query=CHEBI%3A37581
fungal oxidoreductases proven to metabolize malonyl-CoA and linked to protein structure data of any type in the Protein Data Bank	www.uniprot.org/uniprot/?query=annotation%3A%28type%3A%22catalytic+activity%22+inchikey%3ALTYOQGRJFJAKNA-DVVLENMVSA%29+taxonomy%3A%22Fungi+%289FUNG%29+%5B4751%5D%22+goa%3A%28%22oxidoreductase+activity+%5B16491%5D%22%29+reviewed%3Ayes+database%3A%28type%3Apdb%29

## Data Availability

Publicly available datasets were analyzed in this study. This data can be found here: [www.uniprot.org, www.rhea-db.org and www.ebi.ac.uk/chebi].

## Supplementary Materials

The following are available online at https://www.mdpi.com/2218-1989/11/1/48/s1, Table S1: Novel families of enzymes curated in UniProtKB/Swiss-Prot but not specifically represented in InterPro. Figure S1: Sample of the UniProt website simple search tool, Figure S2: Filtering a simple search result on the UniProt website, use of view option, Figure S3: Filtering a simple search result on the UniProt website, restricting search results to suggested organisms, Figure S4: Personalizing the content displayed in each column of the UniProtKB website, Figure S5: Details on result columns of a simple search result on the UniProt website, Figure S6: Downloading the result of a simple search result on the UniProt website, Figure S7: Sample federated SPARQL query to retrieve enzymes known to metabolize compounds identical or similar to patulin, Figure S8: Schematic description of enzyme annotation in UniProtKB.
