# Supplementary material for: Diverse Taxonomies for Diverse Chemistries: Enhanced Representation of Natural Product Metabolism in UniProtKB
Source: Metabolites. 2021 Jan 12;11(1):48. doi: 10.3390/metabo11010048 (PMC7827101; doi:10.3390/metabo11010048)
Supplement: Supplementary file 1 [file metabolites-11-00048-s001.pdf]

Diverse taxonomies for diverse chemistries: enhanced representation of natural product metabolism in UniProtKB

Supplementary Table S1. Novel families of enzymes curated in UniProtKB/Swiss-Prot but not specifically represented in InterPro.

Protein family[a]	Function	Swiss-Prot[b]	Swiss-Prot-Rhea[c]	Link to UniProtKB (query on uniprot.org)
**paxM**	FAD-dependent monooxygenase	77	8	family:"paxm fad-dependent monooxygenase family"
**lovG**	esterase	29	4	family:"lovg family"
**avfA**	oxidoreductase	20	0	family:"avfa family"
**paxB**	terpene cyclase	17	3	family:"paxb family"
**tpcK**	decarboxylase	16	9	family:"tpck family" AND reviewed:yes
**AscI /paxA**	terpene cyclase	12	2	family:"paxa family"
**easN**	O-acetyltransferase	11	2	family:"fumigaclavine b o-acetyltransferase family"
**mdpH-2**	anthrone oxygenase	10	7	family:"anthrone oxygenase family"
**AMT4**	thioesterase	9	0	family:"amt4 thioesterase family"
**asaB**	oxidoreductase	9	0	family:"asab hydroxylase desaturase family"
**OpS7**	oxidoreductase	7	0	family:"oxidoreductase ops7 family"
**bfoA**	acetyltransferase	5	0	family:"bfoa family"
**ltsm**	ND	2	0	family:"ltms family"

[a]The name of one representative member is used to name each family. [b]Number of proteins curated in UniProtKB/Swiss-Prot. [c]Number of UniProtKB/Swiss-Prot proteins with annotated Rhea reactions.

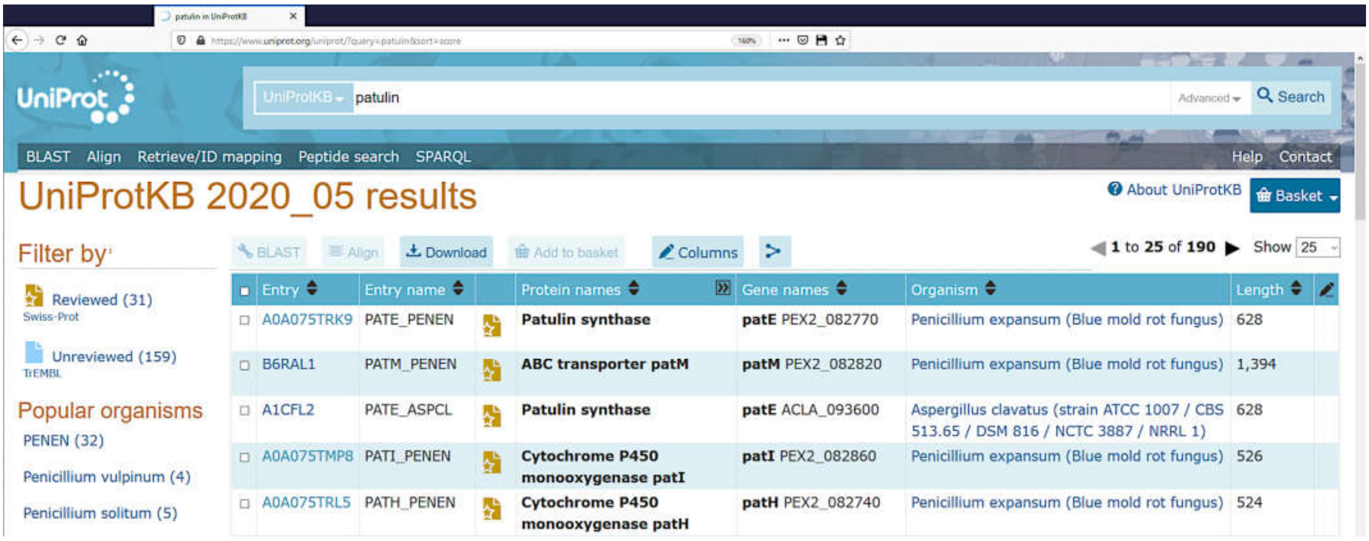

Supplementary Figure S1. Sample of the UniProt website simple search tool. Simple search to mine the UniProtKB content. The request used here was: www.uniprot.org/uniprot/?query=patulin. This leads to the retrieval of UniProtKB entries containing the term “patulin”.

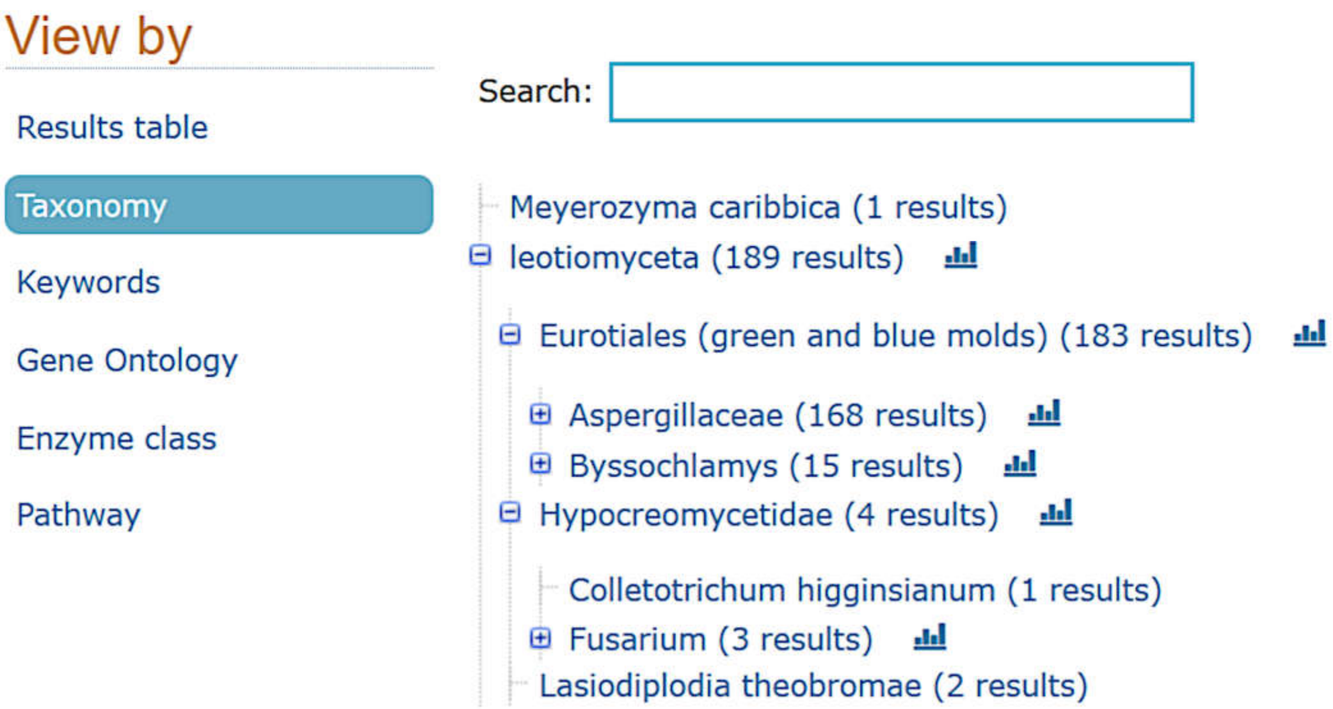

Supplementary Figure S2. Filtering a simple search result on the UniProt website, use of view option. A filter is dedicated to view options following a simple search in UniProtKB. The request used here was: www.uniprot.org/uniprot/?query=patulin. This leads to the retrieval of UniProtKB entries containing the term “patulin”. The filter selected leads to a display by taxonomy, permitting to scroll over the tree of life within the search result.

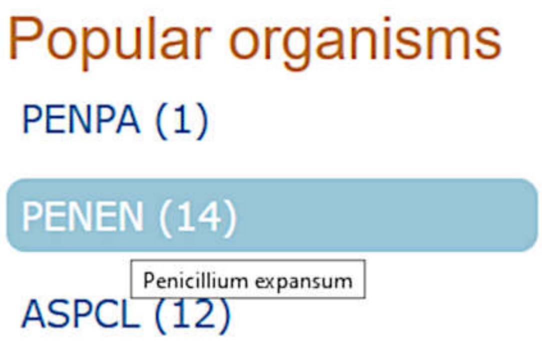

Supplementary Figure S3. Filtering a simple search result on the UniProt website, restricting search results to suggested organisms. A filter is dedicated to popular organism selection following a search in UniProtKB. The request used here was: www.uniprot.org/uniprot/?query=patulin. This leads to the retrieval of UniProtKB entries containing the term "patulin". The filter selected leads to a restriction of the search to *Penicillium expansum* via the "PENEN" option (its mnemonic species identification code www.uniprot.org/help/entry_name).

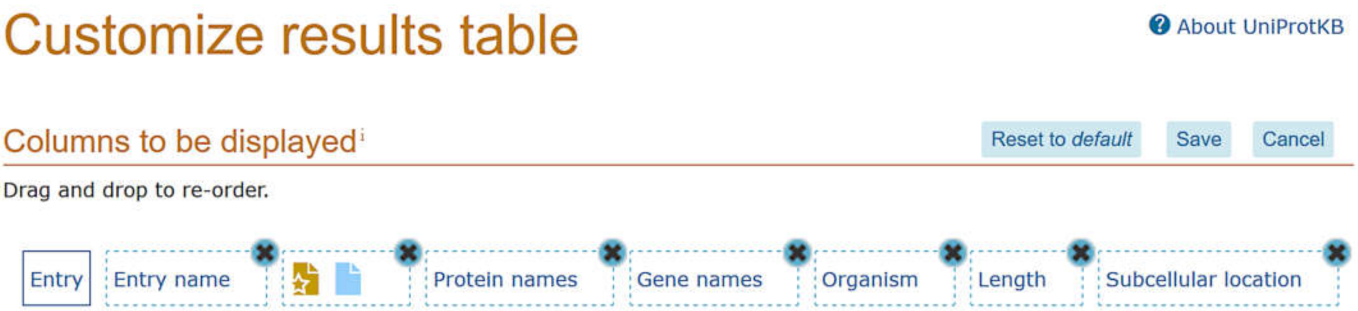

Supplementary Figure S4. Personalizing the content displayed in each column of the UniProtKB website. The custom result table tool of the UniProt website allows to define which to display in columns. Selected columns are "Entry" (www.uniprot.org/help/accession_numbers), "Entry Name" (www.uniprot.org/help/entry_name), "Review status" (Swiss-Prot manually curated in yellow, TrEMBL automatic entries in blue, (www.uniprot.org/help/entry_status), "Protein names" (www.uniprot.org/help/protein_names), "Gene names" (www.uniprot.org/help/gene_name), "Organism" (www.uniprot.org/help/organism-name), "Length" (protein sequence length in amino-acids) and "Subcellular location" (www.uniprot.org/help/subcellular_location_section).

Gene names	Subcellular location [CC]
**patK**<br>PEX2_082880	▪ cytosol 1 Publication
**patI**<br>PEX2_082860	▪ Endoplasmic reticulum membrane 1 Publication ; Single-pass membrane protein 1 Publication
**patB**<br>PEX2_082800	▪ cytosol 1 Publication
**patH**<br>PEX2_082740	▪ Endoplasmic reticulum membrane 1 Publication ; Single-pass membrane protein 1 Publication
**patO**<br>PEX2_082840	▪ Vacuole lumen 1 Publication
**patM**<br>PEX2_082820	▪ Vacuole membrane 1 Publication ; Multi-pass membrane protein 1 Publication<br>▪ Cell membrane 1 Publication ; Multi-pass membrane protein 1 Publication

Supplementary Figure S5. Details on result columns of a simple search result on the UniProt website. Focus on result columns dedicated to gene names and subcellular location following a search in UniProtKB. The request at uniprot.org used here was: pathway:478.918 patulin AND organism:"Penicillium expansum (Blue mold rot fungus) [27334]".

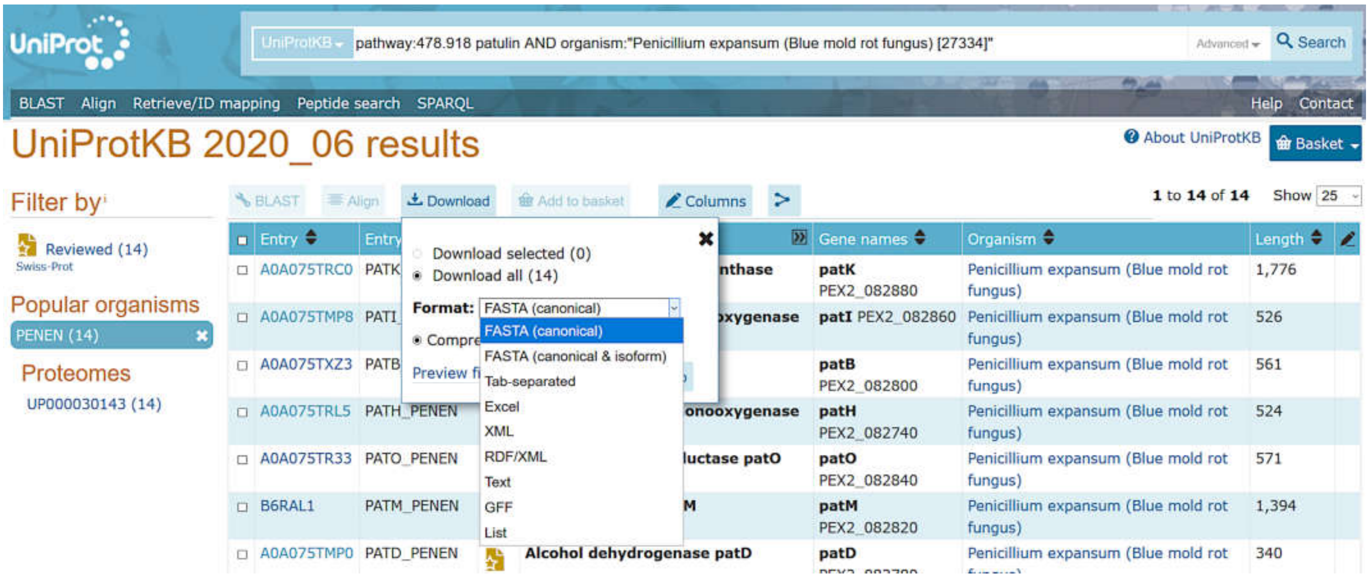

Supplementary Figure S6. Downloading the result of a simple search result on the UniProt website. Various download possibilities following a search in UniProtKB. The request used here was: pathway:478.918 patulin AND organism:"Penicillium expansum (Blue mold rot fungus) [27334]". Displayed columns are "Entry" (www.uniprot.org/help/accession_numbers), "Entry Name" (www.uniprot.org/help/entry_name), "Review status" (Swiss-Prot manually curated in yellow, TrEMBL automatic entries in blue, (www.uniprot.org/help/entry_status), "Protein names" (www.uniprot.org/help/protein_names), "Gene names" (www.uniprot.org/help/gene_name), "Organism" (www.uniprot.org/help/organism-name), "Length" (protein sequence length in amino-acids) and "Subcellular location" (www.uniprot.org/help/subcellular_location_section). The selected download option is FASTA (Canonical) leads to the retrieval of the canonical sequences (www.uniprot.org/help/canonical_and_isoforms) of found entries in the FASTA format, without alternative isoforms.

```
# endpoint: https://sparql.uniprot.org/sparql
#
# query: retrieve ChEBI compounds similar to patulin
#                 - their Rhea reactions
#                 - and their enzymes as annotated in UniProtKB
#
PREFIX rdfs:<http://www.w3.org/2000/01/rdf-schema#>
PREFIX sachem: <http://bioinfo.uochb.cas.cz/rdf/v1.0/sachem#>
PREFIX idsm: <https://idsm.elixir-czech.cz/sparql/endpoint/>
PREFIX up:<http://purl.uniprot.org/core/>
PREFIX rh:<http://rdf.rhea-db.org/>

SELECT ?score ?chebi_compound ?name ?rhea_reaction ?uniprot_enzyme
WHERE {
  SERVICE <https://sparql.rhea-db.org/sparql>{
    #- section 1: idsm/sachem similarity search
    SERVICE <https://idsm.elixir-czech.cz/sparql/endpoint/chebi> {
     [sachem:compound ?chebi_compound; sachem:score ?score]
       sachem:similaritySearch [
       sachem:query "OC1OCC=C2OC(=O)C=C12";
       sachem:cutoff "8e-1"^^xsd:double ;
       sachem:aromaticityMode sachem:aromaticityDetect ;
       sachem:similarityRadius 1 ;
       sachem:tautomerMode sachem:ignoreTautomers
     ] .
  }
  #- section 2: Rhea reaction, to the similar chebi compounds
  ?chebi_compound up:name ?name .
  ?rhea_reaction rh:side/rh:contains/rh:compound/rh:chebi ?chebi_compound .
  }
  #- section 3: UniProt enzymes, catalyzing the Rhea reactions
  ?uniprot_enzyme
up:annotation/up:catalyticActivity/up:catalyzedReaction ?rhea_reaction .
}
ORDER BY DESC(?score)
```

Pseudo code:

```
# Perform a call to the Rhea SPARQL endpoint using "SERVICE"
#- 1: nested SERVICE call to the idsm:chebi endpoint from Rhea
Retrieve ChEBI compounds similar to patulin SMILES using
sachem:similaritySearch with a score threshold (sachem:cutoff) of 0.8
#- 2: from Rhea endpoint
Retrieve Rhea reactions (?rhea_reaction) involving the ChEBI compounds
{?chebi_compound) identified during step 1
#-3: from UniProt endpoint
Retrieve the UniProtKB enzymes (?uniprot_enzyme) annotated with
the ?rhea_reaction identified during step 2
```

Supplementary Figure S7. Sample federated SPARQL query to retrieve enzymes known to metabolize compounds identical or similar to patulin. The query is designed for the UniProt SPARQL endpoint and uses two nested SERVICE to the Rhea and IDSM SPARQL endpoints (see its graphical representation in Figure 5). To see SPARQL in action, simply copy paste the query into sparql.uniprot.org/sparql and run it.

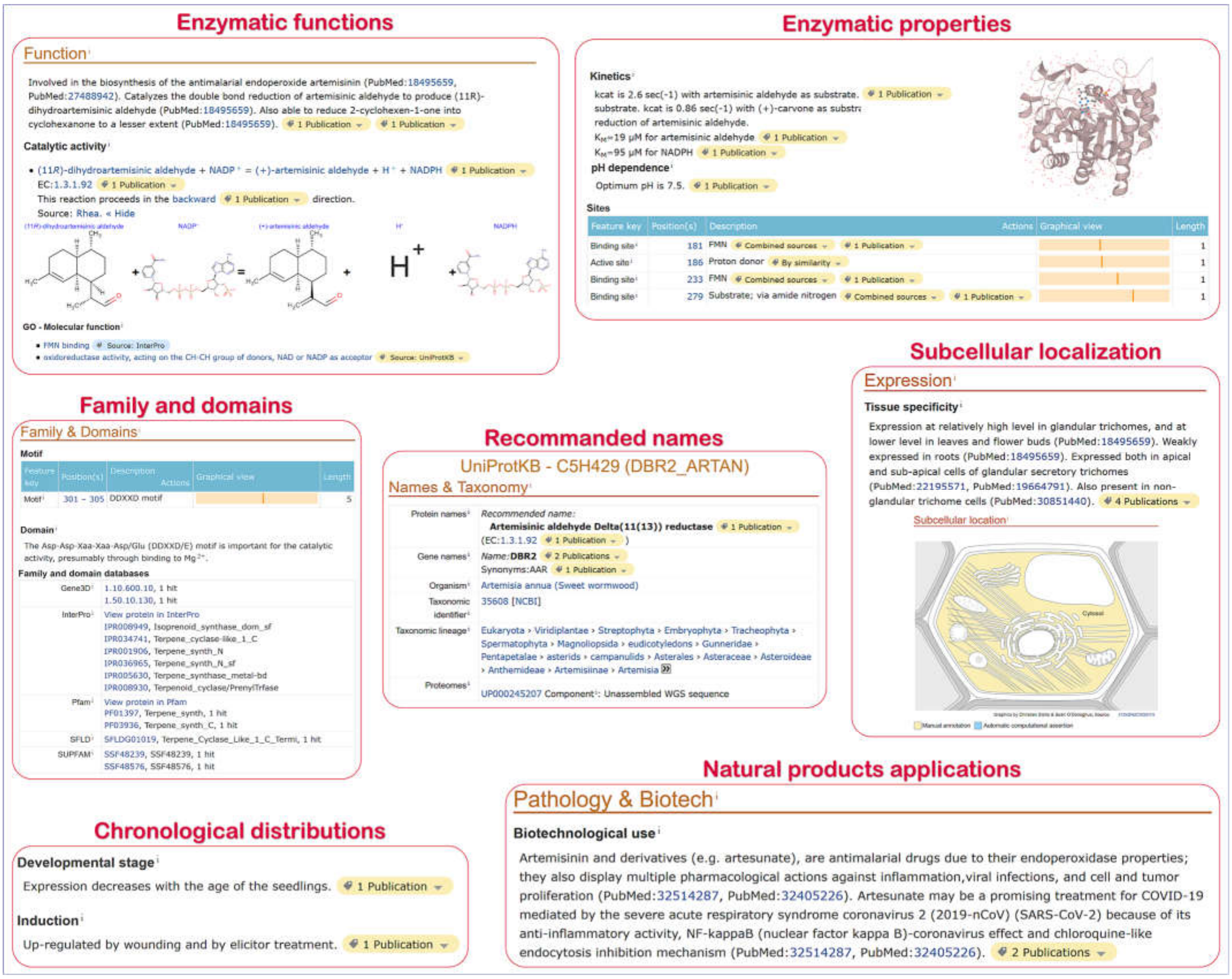

Supplementary Figure S8. Schematic description of enzyme annotation in UniProtKB. All aspects of biology and physical properties publicly available are reported and standardized during expert biocuration. Samples of UniProt.org display are grouped by following topics: “Recommended names”, “Enzymatic function”, “Enzymatic properties”, “Subcellular localization”, “Chronological distributions”, “Family and domains” and “Natural products applications”. Data are extracted from two proteins of *Artemisia annua*, beta-caryophyllene synthase QHS1 (UniProtKB:Q8SA63) and artemisinic aldehyde delta(11(13)) reductase DBR2 (UniProtKB:C5H429). Their complete annotations are available on uniprot.org: www.uniprot.org/uniprot/Q8SA63 and www.uniprot.org/uniprot/C5H429.
